# A Novel Nutraceuticals Mixture Improves Liver Steatosis by Preventing Oxidative Stress and Mitochondrial Dysfunction in a NAFLD Model

**DOI:** 10.3390/nu13020652

**Published:** 2021-02-17

**Authors:** Moris Sangineto, Vidyasagar Naik Bukke, Francesco Bellanti, Rosanna Tamborra, Archana Moola, Loren Duda, Rosanna Villani, Antonino Davide Romano, Gaetano Serviddio

**Affiliations:** 1 C.U.R.E. (University Center for Liver Disease Research and Treatment), Liver Unit, Department of Medical and Surgical Sciences, University of Foggia, 71122 Foggia, Italy; moris.sangineto@unifg.it (M.S.); sagar.niper@gmail.com (V.N.B.); rosanna.tamborra@unifg.it (R.T.); archana.moola@unifg.it (A.M.); rosanna.villani@unifg.it (R.V.); antoninodavide.romano@unifg.it (A.D.R.); 2 Internal Medicine, Department of Medical and Surgical Sciences, University of Foggia, 71122 Foggia, Italy; francesco.bellanti@unifg.it; 3 Pathology Unit, Department of Clinical and Experimental Medicine, University of Foggia, 71122 Foggia, Italy; segreteriamedint@libero.it

**Keywords:** non-alcoholic fatty liver disease, nutraceutical, mitochondria, micronutrients, steatosis, oxidative stress

## Abstract

Non-alcoholic fatty liver disease (NAFLD) is the leading cause of liver disease globally, and represents a health care burden as treatment options are very scarce. The reason behind the NAFLD progression to non-alcoholic steatohepatitis (NASH) is not completely understood. Recently, the deficiency of micronutrients (e.g., vitamins, minerals, and other elements) has been suggested as crucial in NAFLD progression, such that recent studies reported the potential hepatic antioxidant properties of micronutrients supplementation. However, very little is known. Here we have explored the potential beneficial effects of dietary supplementation with FLINAX, a novel mixture of nutraceuticals (i.e., vitamin E, vitamin D3, olive dry-extract, cinnamon dry-extract and fish oil) in a NAFLD model characterized by oxidative stress and mitochondrial function impairment. Steatosis was firstly induced in Wistar rats by feeding with a high-fat/high-cholesterol diet for 4 weeks, and following this the rats were divided into two groups. One group (n = 8) was treated for 2 weeks with a normal chow-diet, while a second group (n = 8) was fed with a chow-diet supplemented with 2% FLINAX. Along with the entire experiment (6 weeks), a third group of rats was fed with a chow-diet only as control. Statistical analysis was performed with Student’s T test or one-way ANOVA followed by post-hoc Bonferroni test when appropriate. Steatosis, oxidative stress and mitochondrial respiratory chain (RC) complexes activity were analyzed in liver tissues. The dietary supplementation with FLINAX significantly improved hepatic steatosis and lipid accumulation compared to untreated rats. The mRNA and protein levels analysis showed that *CPT1A* and *CPT2* were up-regulated by FLINAX, suggesting the enhancement of fatty acids oxidation (FAO). Important lipoperoxidation markers (i.e., HNE- and MDA-protein adducts) and the quantity of total mitochondrial oxidized proteins were significantly lower in FLINAX-treated rats. Intriguingly, FLINAX restored the mitochondrial function, stimulating the activity of mitochondrial RC complexes (i.e., I, II, III and ATP-synthase) and counteracting the peroxide production from pyruvate/malate (complex I) and succinate (complex II). Therefore, the supplementation with FLINAX reprogrammed the cellular energy homeostasis by restoring the efficiency of mitochondrial function, with a consequent improvement in steatosis.

## 1. Introduction

Non-alcoholic fatty liver disease (NAFLD) is a chronic liver disease, whereby the liver fat accumulation exceeds than 5% of the hepatocytes in the absence of alcohol abuse [1,2]. NAFLD encompasses different histological conditions, ranging from simple steatosis (fatty liver), to the non-alcoholic steatohepatitis (NASH). With fibrosis development, NASH can further evolve into liver cirrhosis, a condition potentially complicated by hepatocellular carcinoma (HCC) [3,4]. NAFLD is emerging as a common complication of the metabolic syndrome (MS), a health care burden due to the increasing prevalence of obesity and Type 2 Diabetes Mellitus [5]. NAFLD has become the leading cause of liver disease (25% of the global population) [6], and the second leading indication for liver transplantation in western countries [7]. Unfortunately, there are no compounds efficiently used for treatment, as the pathogenesis of the disease is not completely known yet [8]. NAFLD is characterized by energy expenditure imbalance and metabolic perturbations, which can be fuelled by the assumption of certain macronutrients [9]. For instance, saturated fatty acids (SFA), simple sugars (e.g., sucrose and fructose), trans-fats and animal proteins, by modulating the triglyceride metabolism and the antioxidant activity, affect insulin sensitivity and promote hepatic triglycerides’ accumulation [10]. Therefore, nutrition plays a central role in NAFLD development. However, the contribution of micronutrients has received less attention in the scientific community. Micronutrients are electrolytes, minerals, vitamins, and carotenoids, which are needed in microgram to milligram quantities to sustain physiologic functions [11]. Recently, deficiency in micronutrients has been reported as crucial in NAFLD progression, such that micronutrients-based treatments have been proposed [12,13]. In fact, these molecules exert important functions in regulating redox biology and lipid metabolism, whose perturbations are hallmarks of NAFLD progression. Therefore, in an established rodent model of NAFLD, we investigated whether the supplementation with a new mixture of micronutrients, Flinax (Orga Bio Human Srl, Rome, Italy), was able to exert beneficial effects in terms of steatosis and cellular energy homeostasis. 

## 2. Materials and Methods

### 2.1. Animals and Experimental Design

**Figure 1:**
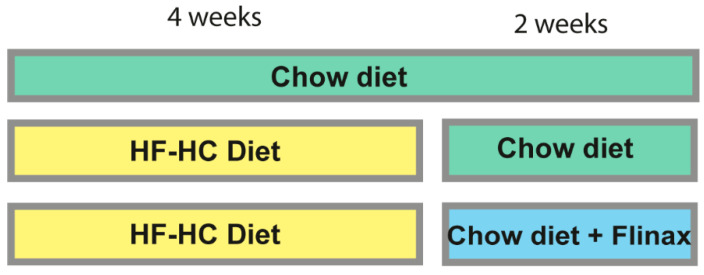
Experimental design. Wistar rats were fed with a high-fat, high-cholesterol diet for 4 weeks in order to induce steatosis. Following this, one group of rats was treated with chow-diet for 2 weeks, while another group was fed with chow-diet supplemented with 2% Flinax. During the entire experiment, a third group of rats was fed with chow-diet only for 6 weeks, serving as the control. HF-HC: high fat high cholesterol.

All animals received care in compliance with national and local law, including ethical approval. Male, 8-week-old Wistar rats (*n* = 22) (Harlan Laboratories, San Pietro al Natisone, Italy) were maintained in individual cages with a 12 h light/12 h dark cycle. All the rats were fed with a high-fat and high-cholesterol diet (HF-HC; 60% cocoa butter + 1.25% cholesterol) ad libitum for 4 weeks in order to induce steatohepatitis. After 4 weeks, rats were divided into two dietary groups: one group (*n* = 8) was fed with chow-diet (HFHCD+CD), while the test group (*n* = 8) was treated with chow-diet supplemented with 2% Flinax (containing vitamin D3, Vitamin E, dry extract of olive, cinnamon dry extract, fish oil) (HFHCD+CD&Flinax) (Table 1) for 2 weeks. Throughout the whole experimentation time, a group of rats (*n* = 6) was fed with chow-diet only (6 weeks) as the control group (referred to as CD) (Figure 1). Diets were prepared by Mucedola Srl (Settimo Milanese, Italy) according to the levels of components previously reported [14]. Rats were weekly weighted, and the amount of food consumed was daily monitored. At the end of the study (6 weeks), after 8 h fasting, animals were anesthetized with Tiletamine/Zolazepam and then sacrificed for the collection of liver samples.

### 2.2. Histology 

Sections of formalin-fixed, paraffin-embedded liver samples were stained with hematoxylin/eosin and blind-analyzed by a pathologist in order to quantify hepatic steatosis. Microscopic analysis of at least 5 randomly chosen high-power magnification fields was used to calculate the percentages of hepatocytes with macrosteatosis and with microsteatosis. The score was elaborated as follows: (% of cells with macrosteatosiX2) + % of cells with microsteatosis.

### 2.3. Triglycerides Analysis

Hepatic triglyceride content was analyzed as previously described [15]. Briefly, frozen liver tissue was homogenized in PBS, adjusting the volume to the weight, and incubated for 30 min at 95 °C. Centrifugation at 12,000× *g* followed, and the triglycerides were measured by absorbance in supernatant incubated with an appropriate reagent (Roche, Switzerland). Fatty-free BSA-coated vials were used to contain the triglycerides suspensions (Sigma, St. Louis, MO, USA).

### 2.4. Isolation of Mitochondria 

Fresh liver tissue was chilled on ice and washed in a medium containing 0.25 M sucrose, 5 mM K-EDTA pH 7.4, 10 mM Tris-HCl pH 7.4 and 0.2% fatty acid-free BSA to remove lipids, blood and connective tissue, and was processed for the preparation of mitochondria as previously reported [16]. Briefly, the tissue was minced by scissors and washed 3 times in the prechilled glass beaker, using the same buffer. Centrifugation steps followed.

### 2.5. Mitochondrial Complexes Activity

For the OXPHOS complexes I, II, III, IV and V, enzymatic activity was measured in isolated mitochondria. A total of 40 µg of mitochondrial protein was used to determine the activity of each complex. The assays were performed at 37 °C in 1 mL of medium as previously reported [17,18]. The volume of mitochondrial extract (40 µg) was previously determined by Bradford assay.

#### 2.5.1. Complex I Assay

In total, 40 μg of each enzyme solution was mixed with complex I buffer (25 mM KH_2_PO_4_ (pH 7.4), 130 μM β-Nicotinamide adenine dinucleotide (NADH), 240 μM potassium cyanide (KCN), 10 μM antimycin A, and 0.1% BSA). The reaction was started by the addition of 50 μM 2,3-dimethoxy-5-methyl-6-n-decyl-1,4-benzoquinone (DB). The change in absorbance at 340 nm was recorded for 3 min. Reference was measured in the presence of 2.5 μM rotenone (dissolved in ethanol). Enzyme activity was calculated with molar extinction coefficient (*ε*) for the NADH (6.22 mM^−1^ cm^−1^).

#### 2.5.2. Complex II Assay

In total, 40 μg of each enzyme solution was incubated with complex II buffer (25 mM KH_2_PO_4_ (pH 7.8), 2 mM EDTA, 1 mg/mL BSA, 10 mM succinate, 1 mM KCN, 4 μM rotenone, and 10 μM antimycin A) for 10 min. After the addition of 50 μM 2,6-Dichlorophenolindophenol (DCPIP), the change in absorbance at 600 nm was recorded for 2 min for reference. The addition of 10 mM malonate inhibits the oxidation of succinate. Enzyme activity was calculated with *ε* for the DCPIP (19.1 mM^−1^ cm^−1^). 

#### 2.5.3. Complex III Assay

In total, 40 μg of each enzyme solution was mixed with complex III buffer (25 mM KH2PO4 (pH 7.8), EDTA 2mM, and 1 mg/mL BSA) with 80 µM decylubiquinol, 240 µM KCN, 4 µM rotenone, 200 µM ATP and 30 μM cytochrome c. The change in absorbance at 550 nm was recorded for 3 min. Cytochrome c was fully reduced at the end of the measurement with dithionite. The reference was measured without enzyme solution. Enzyme activity was calculated with *ε* for the reduced cytochrome c (19.6 mM^−1^ cm^−1^).

#### 2.5.4. Complex IV Assay

Reduced cytochrome c was prepared using sodium dithionite. In total, 40 μg of each enzyme solution was mixed with complex IV buffer (10 mM KH_2_PO_4_, 0.25 M sucrose, and 1 mg/mL BSA). The change in absorbance at 550 nm was recorded for 2 min. Following this, 240 µM KCN was added to fully oxidized cytochrome c at the end of the measurement. The reference was measured without enzyme solution. Enzyme activity was calculated with *ε* for the reduced cytochrome c (19.6 mM^−1^ cm^−1^).

#### 2.5.5. Complex V (ATP-Synthase) Assay

Complex V buffer (50 mM Tris-Hcl (pH 8.0), 5 mg/mL BSA, 20 mM MgCl_2_, 50 mM KCl, 0.2 mM NADH, 10 mM phosphoenolpyruvate, 5 μM antimycin A, 15 μM Carbonylcyanide3-chlorophenylhydrazone), 4 units of lactate dehydrogenase, and pyruvate kinase were incubated with 2.5 mM ATP for 2 min. After this, 40 μg of each enzyme solution was added to the above mixture, and the change in absorbance at 340 nm was recorded for 5 min. The reference was measured in the presence of 3 μM oligomycin for 5 min. Enzyme activity was calculated with ε for the NADH (6.22 mM^−1^ cm^−1^). 

### 2.6. Western Blot Analysis

In total, 40 μg samples of proteins from liver homogenates were loaded in a 10% SDS-PAGE and transferred to a nitrocellulose membrane, blocked for 1.5 h using 5% non-fat dry milk in TBS-t and incubated over night at 4 °C with primary UCP2 antibody (goat polyclonal UCP2 purchased from Santa cruz Biotechnologies, Santa Cruz, CA, USA). Then, the membrane was incubated for 1.5 h with a rabbit HRP-conjugated anti-mouse (Bio-Rad Laboratories Inc., Segrate (MI), Italy). Bands were detected by the ClarityTM Western ECL Blotting Substrate using a ChemiDoc MP system (Bio-Rad Laboratories Inc., Segrate (MI), Italy), and quantified by the Image LabTM Software. 

### 2.7. Gene Expression Analysis by RT-PCR 

RNA was extracted from liver tissue using pure link RNA kit (Ambion) according to the manufacturer’s protocol. Equal amounts of RNA were reverse transcribed to cDNA using a high-capacity cDNA reverse transcription kit (Applied Biosystems) according to the manufacturer’s instructions. Real-time PCR was performed, using Sso Advanced universal SYBR green supermix on a Bio-Rad CFX96 Real-Time system as previously reported [19]. The cycle threshold (Ct) was determined, and the relative gene expression was calculated with the ΔΔCT method. The following primers were used in qPCR assays (Table 2):

### 2.8. Liver Tissue and Mitochondrial HNE and MDA Adducts

4-hydroxy-2-nonenal (HNE) and malondialdehyde (MDA) fluorescent adducts formed with mitochondrial proteins were monitored by spectrofluorimetry as previously reported [20,21]. Briefly, 100 mg of liver tissue is homogenized with 100 µL of 1.15% KCl buffer, treated with 500 µL of 10% TCA and washed with 6 mL Ethanol/ether (3:1) three times. The pellet obtained after centrifugation at 6000 RPM for 5 min is dried and suspended in distilled water. Fluorescent emission was at 460 nm, and excitation was at 390 nm for MDA-adducts and 355 nm for HNE-adducts.

### 2.9. SOD and Catalase Activity

Commercial kits were used to measure superoxide dismutase activity (706002, Cayman Chemical, Ann Arbor, MI, USA) and catalase activity (707002, Cayman Chemical, Ann Arbor, MI, USA) in freshly prepared liver mitochondria according to the manufacturer’s protocols.

### 2.10. Measurement of Mitochondrial H_2_O_2_ Production

The rate of peroxide production was determined in isolated liver mitochondria as previously reported [16]. Briefly, mitochondrial H_2_O_2_ production was measured at 37 °C following the oxidation of Amplex Red by horseradish peroxidase in isolated rat liver mitochondria using 5 mM pyruvate plus 1 mM malate or 5 mM succinate as respiratory substrates. The fluorescence of supernatants was measured using 530 nm as excitation wavelength and 590 nm as emission wavelength. The rate of peroxide production was calculated using a standard curve of H_2_O_2_.

### 2.11. Western Blot Analysis of Hepatic Oxidized Proteins

The analysis of oxidized proteins was performed by western blot in liver mitochondria using an Oxyblot kit (Millipore Bioscience Research Reagents, Temecula, CA, USA) [16]. The same amounts of mitochondrial proteins (35 µg) were reacted with dinitrophenyl hydrazine (DNPH) for 20 min, followed by neutralization with a solution containing glycerol and 2-mercaptoethanol, resolved in 12% SDS-polyacrylamide gel electrophoresis. After the transfer to a nitrocellulose membrane, a blocking step with non-fat milk and incubation with a rabbit anti-DNPH antibody (1: 150) at 4 °C overnight followed. After washing, the membrane was incubated with the secondary antibody (1:300) conjugated to horseradish peroxidase and detected by a chemiluminescence detection kit (Cell Signaling Technology Inc., Danvers, MA, USA). Reactive bands were visualized by the enhanced chemiluminescence method on a VersaDoc Image System (Bio-Rad Laboratories, Hercules, CA, USA). Band density was determined with TotalLab software. The test provides a qualitative analysis of the total proteins’ oxidation state change.

### 2.12. Statistical Analysis

Statistical analysis was performed with GraphPad Prism 8. One-way analysis of variance, followed by post-hoc Bonferroni test, was used to analyze differences between three groups and multiple comparison. Student’s *t* test was used for differences between two groups. Data are shown as mean ± SEM. Statistical significance was considered with *p* < 0.05.

## 3. Results

### 3.1. Flinax Reduced Hepatic Steatosis and Oxidative Stress

**Figure 2:**
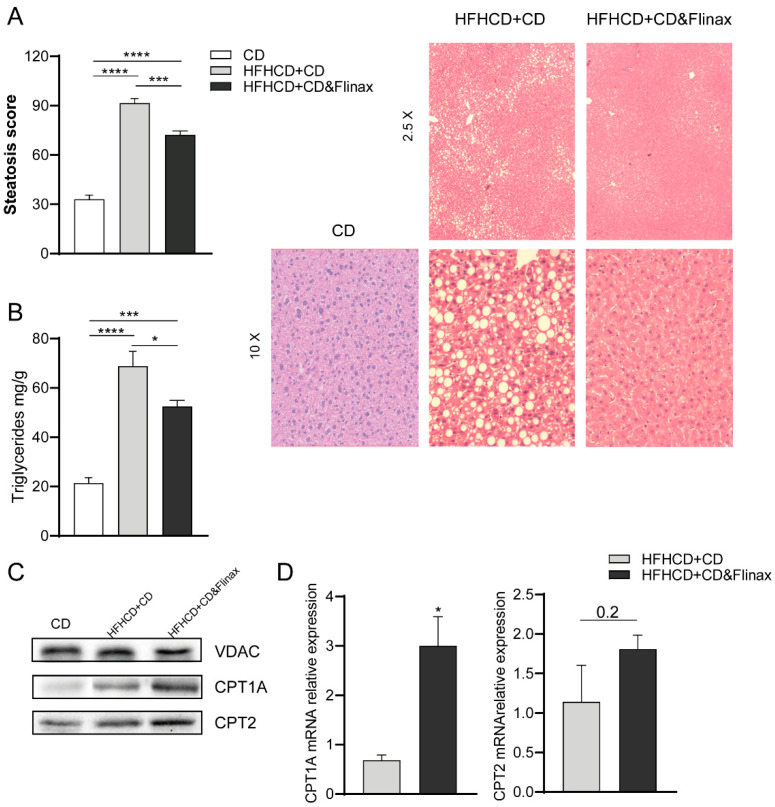
Dietary supplementation with Flinax reduces hepatic steatosis. (**A**) Histological determination of hepatic steatosis with representative pictures of H&E staining (CD = n5; HFHCD+CD = n8; HFHCD+CD&Flinax = n8). (**B**) Liver Triglycerides content (CD = n6; HFHCD+CD = n8; HFHCD+CD&Flinax = n8); (**C**) Protein levels of CPT1A and CPT2 determined by western blot analysis (CD = n4; HFHCD+CD = n8; HFHCD+CD&Flinax = n8); (**D**) CPT1A and CPT2 mRNA expression fold over HFHCD+CD (HFHCD+CD = n6; HFHCD+CD&Flinax = n6). Data are expressed in mean ± SEM; * *p* < 0.05; *** *p* < 0.001; **** *p* < 0.0001 according to Student’s *t* test for comparison between two groups, or one-way ANOVA followed by post-hoc analysis with Bonferroni test for comparison between more groups; CD, chow diet; HFHCD, high-fat, high-cholesterol diet; CPT1A, Carnitine Palmitoyltransferase 1A; CPT2, Carnitine Palmitoyltransferase 2; VDAC, voltage-dependent anion channels.

We investigated the potential beneficial effects of supplementation with Flinax in an established model of NAFLD. To do this, wild type rats were fed for 4 weeks with an HF-HC diet, as we have previously demonstrated that this model is very efficient in inducing fat accumulation and oxidative stress in the liver [18]. Following this, a chow-diet supplemented with 2% of Flinax was administered as the therapeutic for 2 weeks. The daily monitoring of food consumption highlighted that each rat consumed about 0.3 g of Flinax daily. Very interestingly, the Flinax-supplemented rats presented a significantly lower hepatic fat accumulation compared to non-supplemented rats, as shown by histology and triglycerides content (Figure 2A,B). The expression studies did not show differences in important genes involved in FAs synthesis (i.e., SREBP1c, SCD-1, FASN) (Figure S1). In contrast, compared to control CD-fed rats, all HF-HC-fed rats showed a higher production of CPT1A and CPT2 (Figure 2C), two enzymes crucial in FA oxidation (FAO), as they are involved in acylcarnitine and acyl-CoA formation, respectively [22]. However, the increases in mRNA and the protein levels of CPT1A and CPT2 were more significant in Flinax-treated rats (Figure 2C,D). Therefore, we might assert that the excessive fat introduced with the HF-HC diet was contrasted by FAO, and Flinax boosted this adaptive mechanism.

**Figure 3:**
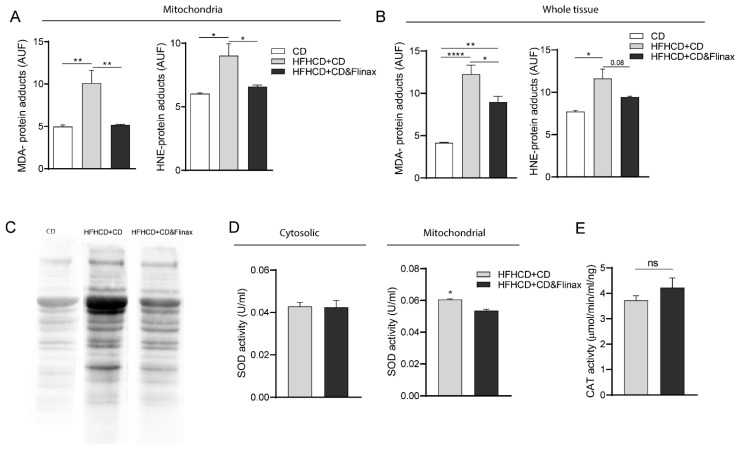
Dietary supplementation with Flinax reduces hepatic oxidative stress. (**A**) Mitochondrial levels of MDA- and HNE-protein adducts (CD = n4–6; HFHCD+CD = n6; HFHCD+CD&Flinax = n6); (**B**) Levels of MDA- and HNE-protein adducts in whole tissue homogenate (CD = n4–6; HFHCD+CD = n6; HFHCD+CD&Flinax = n6); (**C**) Representative picture of the quantity of mitochondrial oxidized proteins detected with Oxyblot (Millipore Bioscience Research Reagents); (**D**) Activity levels of cytosolic and mitochondrial SOD (HFHCD+CD = n8; HFHCD+CD&Flinax = n8); (**E**) Activity level of CAT (HFHCD+CD = n8; HFHCD+CD&Flinax = n8). Data are expressed in mean ± SEM; * *p* < 0.05; ** *p* < 0.01; **** *p* < 0.0001, according to Student’s *t* test for comparison between two groups, or one-way ANOVA followed by post-hoc analysis with Bonferroni test for comparison between more groups; CD, chow diet; HFHCD, high-fat, high-cholesterol diet, Malondialdehyde; HNE, 4-Hydroxynonenal; SOD, superoxide dismutase; CAT, catalase.

In a second step we analyzed the oxidative burden. Notably, the treatment with Flinax significantly reduced the amounts of MDA- and HNE-protein adducts, important lipoperoxidation markers, both measured in isolated mitochondria and whole tissue homogenate (Figure 3A,B). Accordingly, the Oxyblot (Millipore Bioscience Research Reagents) revealed that the total quantity of oxidized proteins in the mitochondria was significantly higher in the untreated rats (Figure 3C). 

The activity of important ROS scavengers such as catalase (CAT) and cytosolic superoxide dismutase (SOD) was not different between the treated and untreated rats (Figure 3D,E). Moreover, in Flinax-supplemented rats the enzymatic activity of mitochondrial SOD was lower (Figure 3D), underlying the fact that in treated rats the induction of scavenging systems was not required. Overall, these results suggested that the administration of Flinax protected the liver, reducing steatosis and oxidative stress. 

### 3.2. Flinax Enhances the Mitochondrial Respiratory Chain Activity

**Figure 4:**
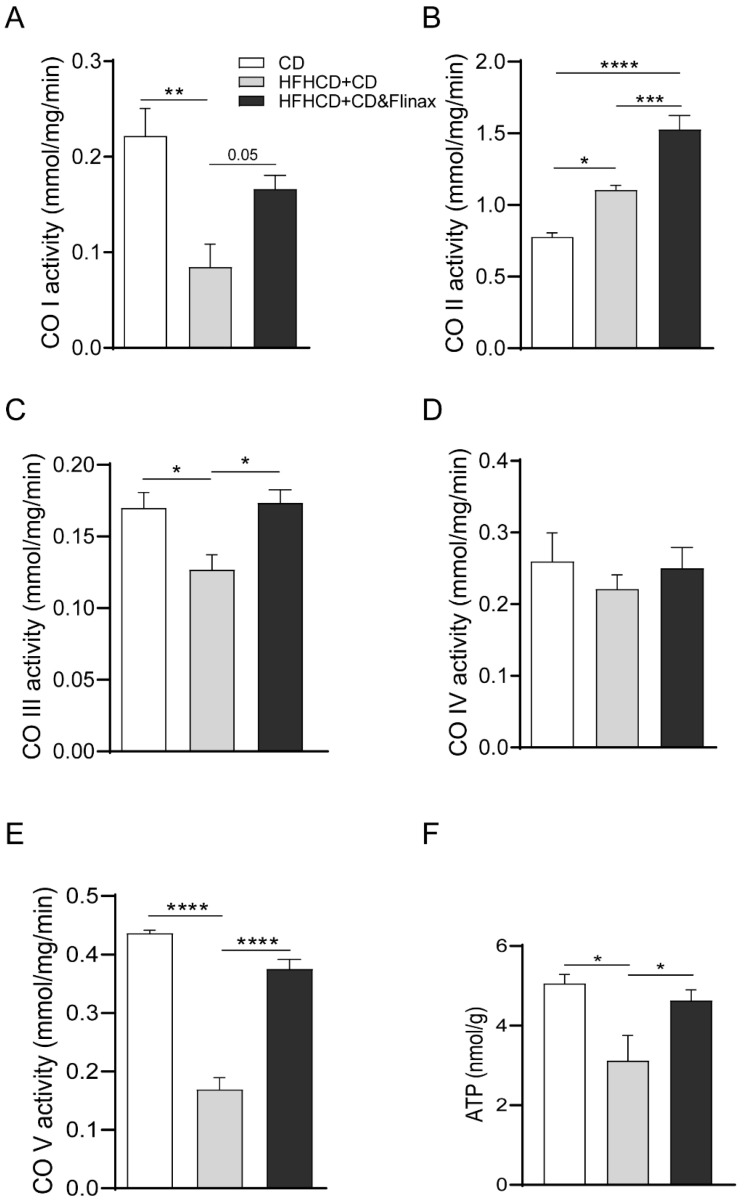
Flinax enhances respiratory chain complexes activity. (**A**) Complex I enzymatic activity; (**B**) Complex II enzymatic activity; (**C**) Complex III enzymatic activity; (**D**) Complex IV enzymatic activity; (**E**) ATP-synthase (Complex V) activity. (CD = n4–6; HFHCD+CD = n8; HFHCD+CD&Flinax = n8). (**F**) ATP content in liver tissue (CD = n4; HFHCD+CD = n6; HFHCD+CD&Flinax = n6). Data are expressed in mean ± SEM; * *p* < 0.05; ** *p* < 0.01; *** *p* < 0.001; **** *p* < 0.0001 according to Student’s *t* test for comparison between two groups, or one-way ANOVA followed by post-hoc analysis with Bonferroni test for comparison between more groups; CD, chow diet; HFHCD, high-fat, high-cholesterol diet; CO, complex.

**Figure 5:**
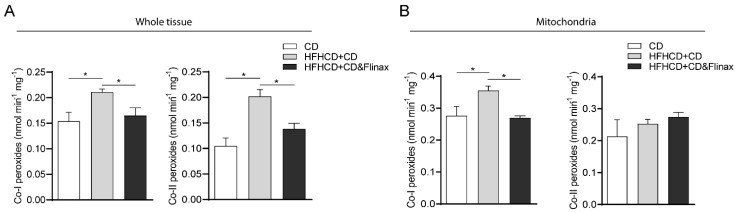
Flinax inhibits peroxide production. (**A**) Peroxide production from pyruvate/malate (complex I) and succinate (complex II), performed in whole tissue homogenate (CD = n4–6; HFHCD+CD = n8; HFHCD+CD&Flinax = n8); (**B**) Peroxide production from pyruvate/malate (complex I) and succinate (complex II), performed in isolated mitochondria (CD = n4–6; HFHCD+CD = n8; HFHCD+CD&Flinax = n8); Data are expressed in mean ± SEM; * *p* < 0.05 according to Student’s *t* test for comparison between two groups, or one-way ANOVA followed by post-hoc analysis with Bonferroni test for comparison between more groups; CD, chow diet; HFHCD, high fat high cholesterol diet; Co-I, complex I; Co-II, complex II.

The imbalance of electron transport chain (ETC) activity in NAFLD has been widely described as a driving force both in humans and in animal models [23]. Therefore, in order to explore the impact of Flinax on mitochondrial metabolism, we analyzed the ETC enzymatic activity and the hydrogen peroxide production rate. Intriguingly, the analysis revealed that the HF-HC diet significantly reduced the activity of complex I and complex III, which was restored by Flinax supplementation (Figure 4A,C). In contrast, the complex II activity was significantly enhanced by the HF-HC diet, and was even more pronounced in Flinax-treated rats (Figure 4B). As the impairment of mitochondrial ATP synthesis has also been described in NASH, we measured the complex V (ATP-synthase) activity and the hepatic ATP content, which were significantly reduced in HF-HCD+CD rats, while Flinax-supplementation restored the normal levels (Figure 4E,F). No difference was found in complex IV activity (Figure 4D). In a previous work we also showed that the HF-HC diet impaired mitochondrial activity, with the consequent production of radicals [18]. To measure this, we quantified the H_2_O_2_ production rate by using pyruvate/malate (complex I) and succinate (complex II) as mitochondrial substrates. Notably, the Flinax significantly reduced the peroxides levels derived from complex I and complex II activity when the analysis was conducted either on whole tissue or in mitochondria (Figure 5A,B).

Along these lines, Flinax recovered an impaired mitochondrial function as shown by the enhancement of respiratory chain activity and mitochondrial ATP production with a lower formation of peroxides.

**Figure 6:**
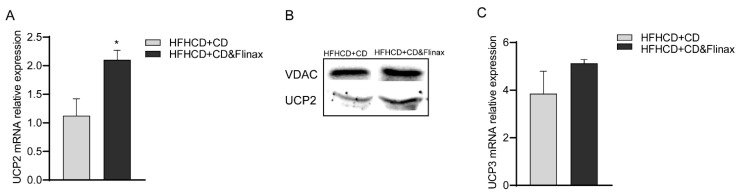
Flinax up-regulates uncoupling proteins. (**A**) mRNA levels of UCP2 fold over HFHCD+CD (HFHCD+CD = n7; HFHCD+CD&Flinax = n6); (**B**) Representative picture of western blot analysis of UCP-2; (**C**) mRNA levels of UCP three-fold over Ctrl (HFHCD+CD=n6; HFHCD+CD&Flinax = n6). Data are expressed in mean ± SEM; * *p* < 0.05 according to the Student’s *t* test. CD, chow diet; HFHCD, high-fat, high-cholesterol diet; UCP2, uncoupling protein 2; UCP3, uncoupling protein 3.

On the other hand, analyzing the expression of uncoupling proteins, we found that the production of uncoupling protein 2 (UCP-2) was higher in the Flinax group, as demonstrated by gene expression and protein levels (Figure 6A,B). UCP-3 also showed a trend towards up-regulation in the Flinax-supplemented rats (Figure 6C). Therefore, despite the positive impact of Flinax on mitochondrial energy homeostasis, we cannot exclude that a certain uncoupling exists, although this did not affect OXPHOS balance.

## 4. Discussion

Although considerable scientific attention is currently addressed to the study of NAFLD, several pathogenic aspects of its development and evolution are still elusive. Therefore, the incomplete mechanistic knowledge and the increasing global incidence make this disease a real health care burden. The complexity of NAFLD pathogenesis has been well described by the multiple hit hypothesis, which highlights the synergistic action of several factors [24]. However, despite the fact that multiple therapeutic approaches have been studied, scarce results have been obtained at the translational level. Recent evidence reported the oxidative imbalance as a cornerstone in the plethora of mechanisms involved in NAFLD [25]. In fact, oxidative stress is already present in simple steatosis, but also behaves as a promoter of inflammatory activation in NASH [26]. Therefore, we used a nutritional model of NAFLD constituted by the administration of an HF-HC diet, as we have recently demonstrated that cholesterol supplementation to the HF diet facilitates mitochondrial disfunction and NAFLD progression [18]. Here, we investigated the potential beneficial effects of a new mixture of micronutrients, named Flinax, containing important molecules that demonstrated antioxidant effects and promising preliminary results in NAFLD patients [13,27,28]. To do this, we used a therapeutic model, whereby NAFLD was induced with HF-HC diet feeding for 4 weeks, followed by 2 weeks of treatment with a chow-diet supplemented with 2% Flinax. This model was used to mimic the clinical condition whereby the patient with NAFLD changes his lifestyle, modifying dietary habits and assuming nutraceuticals. For the study of nutraceuticals, this model would overcome the limitations of classical animal models wherein treatment is administered simultaneously with the high-fat diet. Therefore, the role of supplementation with Flinax was studied and compared to diet change only. Interestingly, the treatment significantly ameliorated steatosis and oxidative stress. Recently, Mosca A. et al. demonstrated that the administration of Vit.E+hydroxytyrosol in children with NAFLD improved steatosis and reduced plasma levels of 4-HNE and 8-OHdG [27]. Here, we found that Flinax significantly reduced the hepatic levels of 4-HNE- and MDA-adducts, and oxidized proteins and peroxides. Therefore, in a second step we investigated whether Flinax impacted the hepatic mitochondrial metabolism by analyzing the activity of ETC complexes. Several studies showed that in the typical lipid-rich condition, the FA overload facilitates the production of oxidants species, which inactivates complex I and III and produces proton leakage [29,30,31,32,33]. Accordingly, we found a reduction in complex I and III activity in untreated rats, while Flinax restored the normal levels. Moreover, as already described in a previous work, the HF-HC diet dampened ATP-synthase (complex V) activity and ATP production in non-supplemented rats. This effect was efficiently contrasted by Flinax administration. Therefore, we may assert that dietary supplementation with Flinax fueled the respiratory chain and contrasted ROS formation, ameliorating cellular energy metabolism an efficiency. Moreover, CPT1A and CPT2, two rate-limiting enzymes for mitochondrial FAO, were up-regulated in untreated rats, and even more so in Flinax-treated rats. Several reports described the increase of FAO in NAFLD as an adaptation to a lipid-rich condition [34]. FAO yields electrons to ETC by mitochondrial complex II, but in NAFLD, some of these electrons leak from complexes I and III to generate ROS [34]. Accordingly, non-supplemented rats showed high FAO (CPT1A and CPT2) and the enhancement of complex II activity, while complex I and III activity decreased. Intriguingly, the supplementation with Flinax fueled even greater FAO and complex II activity, while restoring the high activity of complexes I, III and V. Therefore, the bioenergetics efficiency recovered by Flinax facilitated cellular adaptation mechanisms and avoided ROS formation. However, the increase in UCP proteins underlined that a certain uncoupling probably occurred in Flinax-treated rats. Accordingly, some studies proposed a mild uncoupling as a strategy to prevent mitochondrial ROS formation, as a moderate proton leak would both stimulate oxygen consumption and counteract the reduction of ROS-generating sites [35]. Further studies are needed to indicate the mechanisms behind Flinax-induced mitochondrial adaptation and resistance from external injury, as we might only speculate the role of scavenging activity in preventing ROS formation and the damage of MRC complexes. Moreover, we acknowledge that potential adverse effects have not been investigated in this study. In conclusion, dietary supplementation with FLINAX reprogrammed the cellular energy homeostasis by restoring the efficiency of the mitochondrial function, with the consequent improvement of steatosis.

## Figures and Tables

**Table 1:** Flinax composition (1 g). DHA: docosahexaenoic acid; EPA: eicosapentaenoic acid.

Vitamin D3	25 mcg
Vitamin E	60 mg
Olive dry extract (Olea Europaea) titrated in 20% hydroxytyrosol	15 mg
Cinnamon dry extract (Cinnamomum cassia Presi cortex) titrated in 1% flavonoids	14 mg
Fish oil 56% DHA/EPA	830 mg

**Table 2:** Primers list.

S.No.	Gene	Forward Primer	Reverse Primer
1	*GAPDH*	TCAAGGCTGAGAATGGGAAG	ATGGTGGTGAAGATGCCAGT
2	*SCD1*	TGTTCGTCAGCACCTTCTTG	TCTTGTCGTAGGGGCGATAC
3	*SREBP1*	ATCTGTGAGAAGGCCAGTG	GCGGGCCACAAGAAGTAGA
5	*CPT1*	TTCAAGGTCTGGCTCTACCA	TCCCTCGTGCAAAATAGGTC
6	*CPT2*	GCCTCTCTTGGATGACAGC	CTGGTGTGCTTATTCTGCT
7	*UCP2*	CTTTGAAGAACGGGACAC	TCCTGCTACCTCCCAGAA
8	*UCP3*	ATGAGTTTTGCCTCCATTCG	AATCGGACCTTCACCACATC

## Data Availability

All relevant data are within the manuscript.

## Supplementary Materials

The following are available online at https://www.mdpi.com/2072-6643/13/2/652/s1, Figure S1: Expression of genes involved in fatty acids synthesis. (**A–C**) mRNA levels of SCD-1, SREBP1c and FASN fold over control (Control = n6; Flinax = n6). Data are expressed in mean ± SEM; SCD-1, stearoyl-CoA-desaturase-1; SREBP1c, Sterol regulatory element-binding transcription factor 1-c; FASN, fatty acids synthetase. Control: rats treated with chow-diet; Flinax: rats treated with chow-diet+Flinax. Figure S2: Cytokines gene expression. No difference was observed in the expressions of TNF-α and IL-1β, while IL-6 was not expressed in the model. Control: rats treated with chow-diet; Flinax: rats treated with chow-diet+Flinax.
