# Supplementary material for: A Novel Nutraceuticals Mixture Improves Liver Steatosis by Preventing Oxidative Stress and Mitochondrial Dysfunction in a NAFLD Model
Source: Nutrients. 2021 Feb 17;13(2):652. doi: 10.3390/nu13020652 (PMC7923152; doi:10.3390/nu13020652)
Supplement: Supplementary file 1 [file nutrients-13-00652-s001.pdf]

nutrients

MDPI

Article

A Novel Nutraceuticals Mixture Improves Liver Steatosis by Preventing Oxidative Stress and Mitochondrial Dysfunction in a NAFLD Model

Moris Sangineto [1,†], Vidyasagar Naik Bukke [1,†], Francesco Bellanti [2], Rosanna Tamborra [1], Archana Moola [1], Loren Duda [3], Rosanna Villani [1], Antonino Davide Romano [1] and Gaetano Serviddio [1,*]

[1] C.U.R.E. (University Center for Liver Disease Research and Treatment), Liver Unit, Department of Medical and Surgical Sciences, University of Foggia, 71122 Foggia, Italy; moris.sangineto@unifg.it (M.S.); sagar.niper@gmail.com (V.N.B.); rosanna.tamborra@unifg.it (R.T.); archana.moola@unifg.it (A.M.); rosanna.villani@unifg.it (R.V.); antoninodavide.romano@unifg.it (A.D.R.)

[2] Internal Medicine, Department of Medical and Surgical Sciences, University of Foggia, 71122 Foggia, Italy; francesco.bellanti@unifg.it

[3] Pathology Unit, Department of Clinical and Experimental Medicine, University of Foggia, 71122 Foggia, Italy; segreteriamedint@libero.it

* Correspondence: gaetano.serviddio@unifg.it; Tel.: +39-0881-736007

† These authors contributed equally to this work.

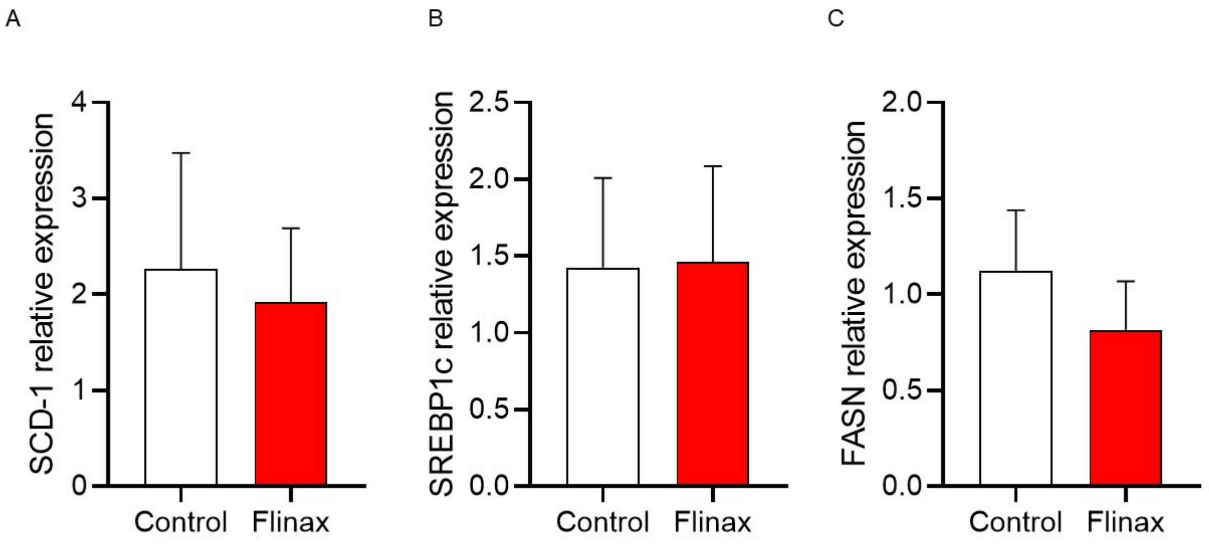

Figure S1. Expression of genes involved in fatty acids synthesis. (**A**–**C**) mRNA levels of SCD-1, SREBP1c and FASN fold over control (Control = n6; Flinax = n6). Data are expressed in mean ± SEM; SCD-1, stearoyl-CoA-desaturase-1; SREBP1c, Sterol regulatory element-binding transcription factor 1-c; FASN, fatty acids synthetase. Control: rats treated with chow-diet; Flinax: rats treated with chow-diet+Flinax.

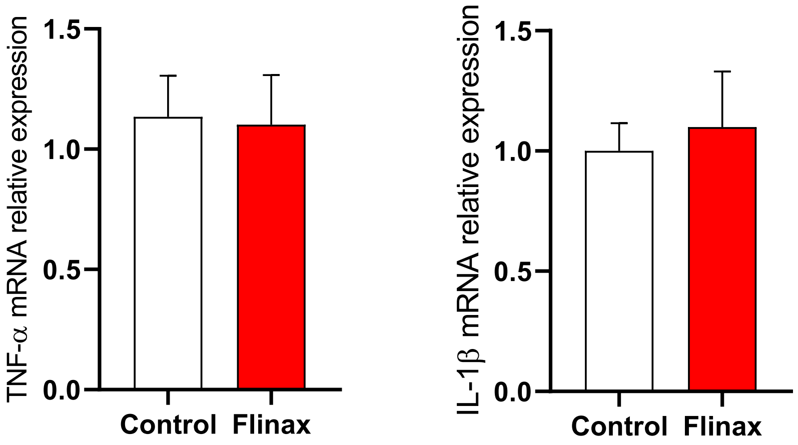

Figure S2. Cytokines gene expression. No difference was observed in the expressions of TNF-α and IL-1β, while IL-6 was not expressed in the model. Control: rats treated with chow-diet; Flinax: rats treated with chow-diet+Flinax.
